# Association of living alone and living alone time with hypertension among Chinese men aged 80 years and older: a cohort study

**DOI:** 10.3389/fpubh.2023.1274955

**Published:** 2024-01-05

**Authors:** Xiang Wang, Miao Dai, Jingsong Xu

**Affiliations:** ^1^Department of Cardiology, The Second Affiliated Hospital of Nanchang University, Nanchang, China; ^2^Department of Cardiology, Jiujiang First People’s Hospital, Jiujiang, China; ^3^Department of Geriatrics, Jiujiang First People’s Hospital, Jiujiang, China

**Keywords:** living alone, living alone time, hypertension, oldest-old men, cohort study

## Abstract

**Objective:**

There is little evidence of the influence of living alone on hypertension risk among men 80 years or older. Additionally, the influence of living alone duration on hypertension risk lacks thorough investigation. Hence, this cohort study examines living alone and its duration’s link to hypertension risk in this specific group.

**Methods:**

We included 2009 older men aged ≥80 years without hypertension from the Chinese Longitudinal Healthy Longevity Survey in the 2008 wave. Follow-up was conducted in the 2011 wave. Multivariable Cox proportional hazards models estimated hazard ratios (HRs) and 95% confidence intervals (CIs) to assess hypertension risk related to living alone and living alone time.

**Results:**

We included 2,009 older men, with a mean age of 90.7 years (standard deviation: 6.8). Over a median follow-up of 2.9 (1.3–3.0) years, 573 participants (28.5%) developed hypertension. Living alone was significantly associated with a higher hypertension risk than living with family (HR: 1.42; 95% CI 1.11–1.80). When compared to living with family, the hypertension risk was increased in the first quartile of living alone time (0–6.1 years) (HR: 1.76; 95% CI 1.16–2.66), the second quartile (6.1–10.6 years) (HR: 1.56; 95% CI 1.07–2.29), and the third quartile (10.6–19.3 years) (HR: 1.66; 95% CI 1.08–2.55). Surprisingly, no significant association was found in the fourth quartile (≥19.3 years) with hypertension risk. Stratified and Interaction analyses indicated no significant interaction effects between subgroups. Sensitivity analyses yielded consistent results.

**Conclusion:**

Living alone was independently associated with an increased risk of hypertension in older men. The highest risk was found in those with the least time alone. These findings imply that social isolation and lack of companionship could be pivotal in hypertension development. Furthermore, the study highlights the need to consider living alone duration when assessing its impact on health outcomes.

## Introduction

Hypertension, or high blood pressure, is a prevalent and significant public health issue among older adults worldwide, posing a considerable burden on healthcare systems and leading to various adverse health outcomes, including stroke (1), heart failure (2), renal diseases (3), and mortality (4). The aging process is often associated with physiological changes that can contribute to the development of hypertension, making older individuals, especially those over 80 years of age, particularly susceptible to this condition (5). In China, the reported prevalence of hypertension among individuals 80 years and older was about 66.7% (6). Consequently, identifying modifiable risk factors associated with hypertension in this specific population is of utmost importance to reduce the incidence of cardiovascular events and improve overall health outcomes.

Several factors contribute to the development and progression of hypertension, including genetic predisposition (7), lifestyle behaviors (8), and comorbidities such as obesity (9), diabetes (10), and chronic kidney disease (11). One factor that has garnered increasing attention in recent years is the living arrangements of older individuals, specifically the prevalence of living alone. The number of older adults living alone has been steadily rising (12), attributed to various societal changes, such as changes in family structure and increased life expectancy (13). While living alone can offer autonomy and independence, it may also lead to social isolation, reduced social support, and increased psychological distress (14). These psychosocial factors, in turn, may contribute to adverse health outcomes, including the development and exacerbation of cardiovascular diseases such as hypertension (15). Previous research has reported mixed findings regarding the association between living alone and hypertension risk, with some studies suggesting a positive association (16, 17), while others indicating no significant association (18) or negative association (19, 20). However, prior studies on living arrangements and hypertension in older adult primarily encompassed broader age groups, neglecting a specific focus on men aged 80 and above. Despite the worldwide surge in hypertension cases, treatment and control rates remain notably low, particularly among men (21). Furthermore, given the disparity in longevity between men and women, the older female population is typically larger (22). Men may face distinct health issues and risks compared to women (23, 24). Consequently, the research focusing on older men aims to address a potentially more vulnerable subgroup within this demographic, recognizing that health outcomes and risk factors can vary by gender. Emphasizing men allows for an exploration of gender-specific associations between living alone and hypertension, with potential implications for tailored public health policies and interventions in reducing hypertension-related health issues in this population. Moreover, the duration of living alone has been proposed to be an important factor in understanding its impact on health outcomes (25). Long-term living alone may have different health implications compared to recent transitions to living alone. However, the influence of living alone time on hypertension risk in older adults has not been explored. Understanding whether prolonged periods of living alone have a greater impact on hypertension risk compared to recent or temporary living-alone situations can inform the development of tailored interventions and support systems.

Therefore, this cohort study seeks to address these gaps in the literature by examining the association between living alone, living alone time, and the risk of developing hypertension in a cohort of community-dwelling older men aged 80 years and above, using data from the Chinese Longitudinal Healthy Longevity Survey (CLHLS). Our research hypothesis is that prolonged periods of living alone among older men may be positively correlated with an increased risk of developing hypertension, given the potential social and lifestyle factors associated with living alone.

## Methods

### Study design and participants

The CLHLS is an ongoing nationwide, population-based study aimed at investigating the factors influencing longevity among older adults in mainland China. This survey was conducted from 1998 to 2018, covering eight waves with 2–3 year intervals between each wave. To collect data, the CLHLS utilized a multi-stage disproportionate and targeted random sampling method, reaching older adults aged 65 and over, as well as their sons and daughters aged 35 to 64, across 23 out of the 31 provinces of China. Each centenarian, identified by a pre-assigned random code, was paired with a nearby octogenarian and a nonagenarian of matching age and gender. The term ‘nearby’ denotes individuals residing in the same village, on the same street, or within the same town, county, or city when applicable. This sampling approach was meticulously crafted to guarantee an equitable representation of randomly selected male and female octogenarians and nonagenarians within the age range of 80 to 99. The sampling frame employed cross-sectional age-sex-residence-specific sampling weights, which were generated at the person-year level and calculated based on the population counts of older adults categorized by age, sex, and rural–urban residence. The survey gathered comprehensive data on various aspects, including general information about the participants and their families, family structure, daily activity ability, self-assessment of health-related quality of life, psychological characteristics, social status, economic status, lifestyle, disease profile, and other aging-related information. To address attrition due to death and loss of follow-up, new participants were enrolled during the follow-up period. The surveys were conducted by trained interviewers in the participants’ homes using a structured questionnaire. More information about the CLHLS survey has been previously published (26).

**Figure 1:**
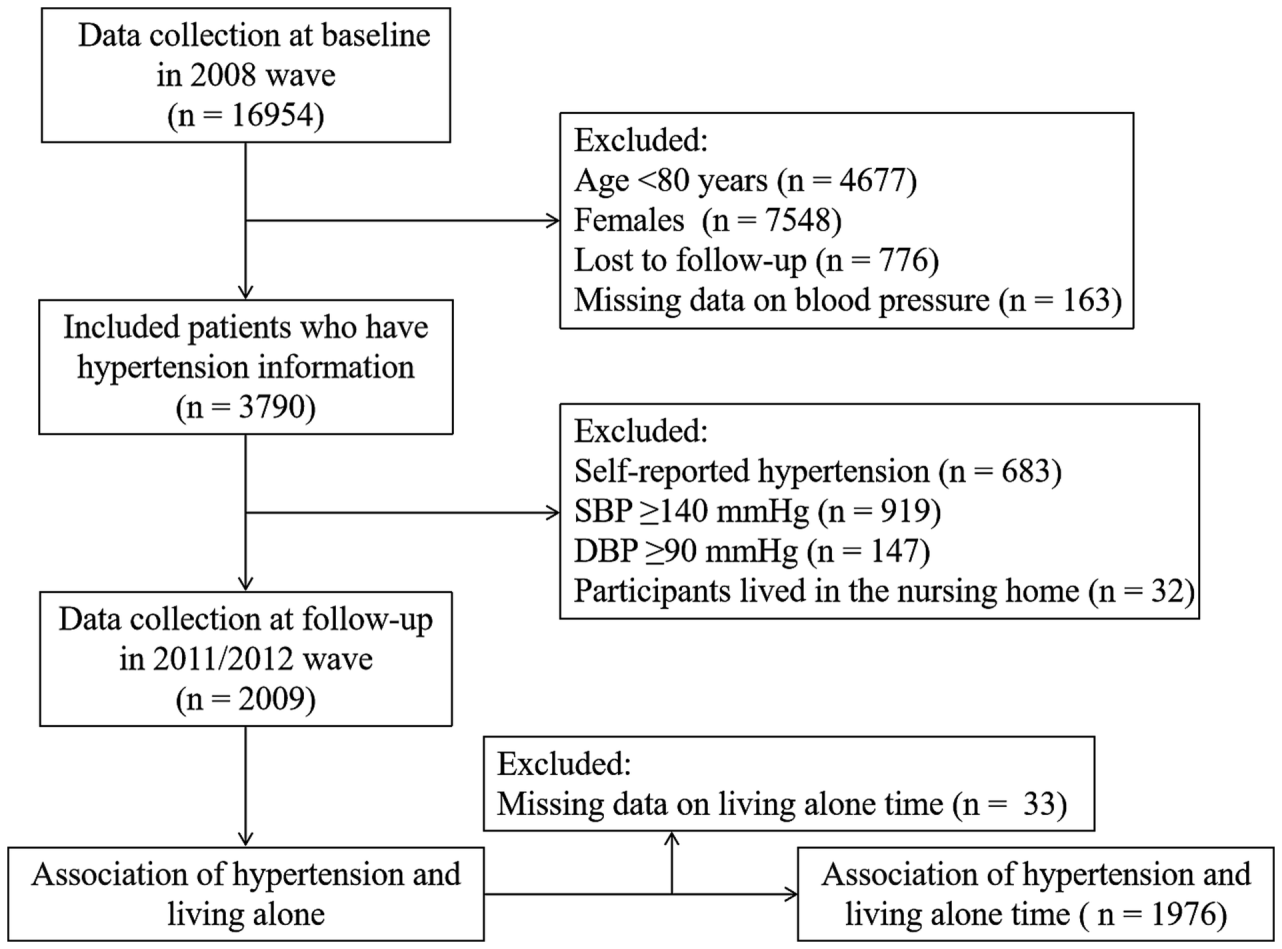
Flowchart of the included study population.

The current study employed data from the 2008 wave due to the lack of two blood pressure measurements in the preceding waves. The follow-up study was conducted during the 2011/2012 wave. This involved surveying a total of 16,954 participants. Participants who were below 80 years old (*n* = 4,677), identified as females (*n* = 7,548), lost to follow-up (*n* = 776), had missing blood pressure information (*n* = 163), had physician-diagnosed hypertension (*n* = 683), had systolic blood pressure (SBP) ≥ 140 mmHg and/or diastolic blood pressure (DBP) ≥ 90 mmHg (*n* = 1,066), and lived in nursing homes (*n* = 32) were excluded from the analysis. Finally, a cohort of 2,009 participants without hypertension at baseline was included in the study to analyze the association of living alone with incident hypertension. For the analysis of the association between living alone time and hypertension, we further excluded participants with missing data on living alone time (*n* = 33). Figure 1 provides a detailed description of the inclusion and exclusion process.

### Living alone and living alone time assessment

At baseline, trained personnel assessed participants’ living arrangements by asking, “Who are you currently living with?” Participants were classified into two groups based on their living arrangements: living alone or living with family. Additionally, participants’ duration of living alone was determined at baseline by inquiring, “When did you commence living alone?” Living alone time was defined as the duration, in years, that an individual had continuously lived alone until the baseline assessment. Subsequently, living alone time was divided into quartiles: Quartile 1 (0–6.1 years), Quartile 2 (6.1–10.6 years), Quartile 3 (10.6–19.3 years), and Quartile 4 (≥ 19.3 years). These quartile categories were established to explore potential dose–response relationships between living alone time and the risk of hypertension.

### Outcome assessment

Blood pressure measurements were taken at baseline and during the follow-up visit. Systolic blood pressure (SBP) and diastolic blood pressure (DBP) were recorded in millimeters of mercury (mmHg) using a mercury sphygmomanometer (upper arm type; Yuyue, Jiangsu, China). Participants seated and relaxed for at least five minutes before measurement, ensuring there was at least a 1-min interval between consecutive blood pressure readings. The average of two consecutive measurements was calculated and used for analysis.

For survivor respondents, participants were classified as having hypertension if they met any of the following criteria: SBP ≥140 mmHg, DBP ≥90 mmHg, or a self-reported history of hypertension diagnosed by a physician (5). For deceased participants, the hypertension status was ascertained through close relatives using the following question: “Did the participant suffer from hypertension before passing away?”

### Covariates

We incorporated baseline sociodemographic characteristics, lifestyle behaviors, and health status into the model. These specific covariates were preselected as potential confounders based on existing literature (8, 27–30). Sociodemographic characteristics comprised age (continuous), years of education (0 years, 1–6 years, or > 6 years), residence (urban or rural), marital status (married or other [widowed, separated, divorced, or never married]), and economic status (independence or dependence). Lifestyle behaviors were assessed based on smoking status (never, past, or current), drinking status (never, past, or current), regular exercise (never, past, or current), frequency of vegetable intake (daily, quite often, occasionally, rarely, or none), frequency of fruit intake (daily, quite often, occasionally, rarely, or none), frequency of meat intake (daily, weekly, monthly, occasionally, rarely, or none), frequency of fish intake (daily, weekly, monthly, occasionally, rarely, or none), and frequency of egg intake (daily, weekly, monthly, occasionally, rarely, or none). Health status consisted of activity of daily living (ADL) limitation (yes or no), body mass index (BMI, underweight [< 18.5 kg/m^2^], normal [18.5–23.9 kg/m^2^], overweight [24–27.9 kg/m^2^], or obese [≥ 28 kg/m^2^]) (31), sleep time (<6 h, 6 to 9 h, or ≥ 9 h), number of natural teeth (0, 1–9, 10–19, or ≥ 20 teeth), and common chronic diseases (self-reported diabetes, heart disease, cerebrovascular disease, and respiratory disease). We defined ADL limitation as experiencing difficulty or requiring assistance with at least one of six daily activities, including bathing, dressing, indoor transferring, toileting, eating, and continence, due to health problems in the previous 6 months (32).

## Statistical analysis

Descriptive statistics were used to summarize the characteristics of the study population according to their living arrangements. For quantitative variables that followed a normal distribution, the means and standard deviations (SDs) were reported. For non-normally distributed quantitative variables, the medians along with the interquartile range were provided. Categorical variables were presented as frequencies and percentages. The multiple imputation method based on the chained equation approach was used to address the missing data, which ranged from 0.3% to 1.3% (Supplementary Table S1). Five repetitions were performed for the imputation process.

Participants who developed hypertension during the follow-up period, as well as those who had hypertension prior to death, were categorized into the hypertension outcome group. All other participants, whether they were alive or deceased, were placed in the non-hypertension outcome group. Participants were censored at the time of the end of the study period or death, whichever occurred first. The follow-up time (in months) was calculated as the time from baseline to end of the follow-up period, or death, whichever occurred first. Since the sampling weight variable in the publicly released CLHLS dataset is derived solely from the age, sex, and urban/rural residence-specific population distribution, without accounting for other crucial compositional factors like living alone and hypertension, we refrained from utilizing sample weights in our main analyses. The crude incidence rates (IR) of hypertension per 100 person-years were calculated for each group. Proportional hazard assumptions were assessed using Schoenfeld residuals. The assumptions of proportionality were met. The association between living alone and living alone time and hypertension risk was assessed using multivariable Cox proportional hazards models, with hazard ratios (HRs) and 95% confidence intervals (CIs) estimated. The models were adjusted for potential confounders, including age, education, residence, marital status, economic status, drinking status, smoking status, regular exercise, ADL limitation, sleep time, number of natural teeth, BMI, diabetes, heart disease, cerebrovascular disease, respiratory disease, frequency of vegetable intake, frequency of fruit intake, frequency of meat intake, frequency of fish intake, and frequency of egg intake. We performed a linear trend analysis by considering the quartiles of living alone time as continuous variables, using the median values from each quartile. We performed a restricted cubic spline analysis, utilizing three knots positioned at the 10th, 50th, and 90th percentiles of alterations in the distribution of living alone time. This analysis aimed to explore the dose–response correlation between changes in this variable and the likelihood of developing hypertension. The reference value for living alone time was established at 0 years. Additional subgroup and interaction analyses were conducted to explore potential effect modifiers, such as education, residence, marital status, economic status, drinking status, smoking status, regular exercise, ADL limitation, number of natural teeth, and BMI. Potential interactions between covariates and living arrangements were tested using cross-product terms.

We conducted sensitivity analyses to check the robustness of the results. First, we conducted a complete case analysis to assess the impact of missing data. Second, we excluded individuals with hypertension during the initial 1-year follow-up period to mitigate potential confounding effects arising from short-term follow-up, considering the chronic nature of hypertension development. Third, participants with pre-existing diabetes, heart disease, or cerebrovascular disease at baseline were excluded to ensure the findings remained unaffected by chronic comorbidities. Lastly, to mitigate potential selection bias, we applied age, sex, and residence-specific weights provided by the CLHLS to ensure the representativeness of our findings for the Chinese population aged 80 years or older.

All statistical analyses were performed using R statistical software version 4.2.2 (R Foundation for Statistical Computing), with a significance level set at *p* value <0.05.

## Results

### Baseline characteristics

Among the 2,009 participants, 328 (16.3%) participants were living alone. The mean age of the participants was 90.7 years (SD: 6.8). Detailed baseline characteristics of the participants are presented in Table 1. In comparison to participants living with family, those who lived alone exhibited a higher likelihood of being younger, residing in rural areas, being unmarried (divorced, widowed, or never married), experiencing economic dependence, not participating in regular exercise, having no limitations in ADL, having inadequate intake of fruits, vegetables, meat, fish, and eggs, as well as demonstrating a lower prevalence of cerebrovascular disease.

**Table 1:** Demographic and clinical characteristics of the study population.

Characteristics	Overall (*n* = 2,009)	Living with family (*n* = 1,681)	Living alone (*n* = 328)	*p* value
Age (years), median (IQR)	90.00 (85.0, 95.0)	90.00 (86.0, 95.0)	90.00 (85.00, 94.00)	<0.001
Rural area, no. (%)	1,260 (62.7)	1,027 (61.1)	233 (71.0)	0.001
Married, no. (%)	739 (36.8)	727 (43.2)	12 (3.7)	<0.001
Education (year), no. (%)				0.596
0	912 (45.4)	755 (44.9)	157 (47.9)	
1–6	851 (42.4)	717 (42.7)	134 (40.9)	
>6	246 (12.2)	209 (12.4)	37 (11.3)	
Economic independence, no. (%)	538 (26.8)	475 (28.3)	63 (19.2)	0.001
Smoking status, no. (%)				
Never	868 (43.2)	718 (42.7)	150 (45.7)	0.178
Current	585 (29.1)	484 (28.8)	101 (30.8)	
Former	556 (27.7)	479 (28.5)	77 (23.5)	
Drinking status, no. (%)				
Never	1,015 (50.5)	857 (51.0)	158 (48.2)	0.640
Current	555 (27.6)	461 (27.4)	94 (28.7)	
Former	439 (21.9)	363 (21.6)	76 (23.2)	
Regular exercise, no. (%)				
Never	1,118 (55.6)	915 (54.4)	203 (61.9)	0.011
Current	608 (30.3)	514 (30.6)	94 (28.7)	
Former	283 (14.1)	252 (15.0)	31 (9.5)	
ADL limitation, no. (%)	366 (18.2)	345 (20.5)	21 (6.4)	<0.001
Sleep time (h), no. (%)				
<6	191 (9.5)	154 (9.2)	37 (11.3)	0.205
6–9	927 (46.1)	789 (46.9)	138 (42.1)	
≥9	891 (44.4)	738 (43.9)	153 (46.6)	
Number of natural teeth, no. (%)				
0–9	1,473 (73.3)	1,228 (73.1)	245 (74.7)	0.796
10–20	324 (16.1)	275 (16.4)	49 (14.9)	
≥20	212 (10.6)	178 (10.6)	34 (10.4)	
BMI (kg/m^2^), no. (%)				
Underweight (<18.5)	710 (35.3)	592 (35.2)	118 (36.0)	0.250
Normal (18.5–4)	1,118 (55.6)	928 (55.2)	190 (57.9)	
Overweight (24–28)	152 (7.6)	135 (8.0)	17 (5.2)	
Obese (≥28)	29 (1.4)	26 (1.5)	3 (0.9)	
Frequency of fruit intake, no. (%)				
Daily	240 (11.9)	224 (13.3)	16 (4.9)	<0.001
Quite often	432 (21.5)	367 (21.8)	65 (19.8)	
Occasionally	807 (40.2)	678 (40.3)	129 (39.3)	
Rarely or none	530 (26.4)	412 (24.5)	118 (36.0)	
Frequency of vegetable intake, no. (%)				
Daily	1,136 (56.5)	978 (58.2)	158 (48.2)	<0.001
Quite often	585 (29.1)	488 (29.0)	97 (29.6)	
Occasionally	229 (11.4)	172 (10.2)	57 (17.4)	
Rarely or none	59 (2.9)	43 (2.6)	16 (4.9)	
frequency of meat intake, no. (%)				
Daily	600 (29.9)	535 (31.8)	65 (19.8)	<0.001
Weekly	774 (38.5)	648 (38.5)	126 (38.4)	
Monthly	223 (11.1)	176 (10.5)	47 (14.3)	
Occasionally	204 (10.2)	159 (9.5)	45 (13.7)	
Rarely or none	208 (10.4)	163 (9.7)	45 (13.7)	
frequency of fish intake, no. (%)				
Daily	141 (7.0)	121 (7.2)	20 (6.1)	0.004
Weekly	636 (31.7)	552 (32.8)	84 (25.6)	
Monthly	380 (18.9)	322 (19.2)	58 (17.7)	
Occasionally	372 (18.5)	310 (18.4)	62 (18.9)	
Rarely or none	480 (23.9)	376 (22.4)	104 (31.7)	
frequency of egg intake, no. (%)				
Daily	683 (34.0)	606 (36.0)	77 (23.5)	<0.001
Weekly	717 (35.7)	599 (35.6)	118 (36.0)	
Monthly	254 (12.6)	192 (11.4)	62 (18.9)	
Occasionally	193 (9.6)	160 (9.5)	33 (10.1)	
Rarely or none	162 (8.1)	124 (7.4)	38 (11.6)	
Diabetes, no. (%)	35 (1.7)	29 (1.7)	6 (1.8)	1.000
Heart disease, no. (%)	102 (5.1)	88 (5.2)	14 (4.3)	0.554
Cerebrovascular disease, no. (%)	107 (5.3)	99 (5.9)	8 (2.4)	0.016
Respiratory disease, no. (%)	273 (13.6)	236 (14.0)	37 (11.3)	0.213

ADL, activity of daily living; BMI, Body Mass Index. Differences in characteristics were compared using the χ^2^ test for categorical variables and the Mann–Whitney U test for continuous variables.

With a median follow-up period of 2.8 years (interquartile range: 1.3 to 3.0, 4436.6 person-years), 573 (28.5%) hypertension was identified. Participant baseline characteristics, categorized based on the development of hypertension during the follow-up period, are presented in Supplementary Table S2. In comparison to older adults without hypertension, those who developed hypertension were more likely, at baseline, to be younger, unmarried (either divorced, widowed, or never married), literate, economically independent, engaged in regular exercise, free from limitations in ADL, and possessed a greater number of natural teeth. Furthermore, they were also more likely to fall into either the overweight or normal weight categories (Supplementary Table S2).

### Association between living alone and living alone time and hypertension risk

The crude IR of hypertension was higher among men living alone (IR per 100 person-years: 15.7) compared to those living with family (IR 100 person-years: 12.4) (Table 2). In the unadjusted analysis, self-reported living alone exhibited a positive association with an increased risk of hypertension in comparison to living with family. Both in model 1 and model 2, the HR and the corresponding 95% CI remained unchanged. In model 3, the relationship between living alone and the risk of hypertension exhibited a similar trend, with a slightly enhanced effect size (HR: 1.42, 95% CI: 1.11–1.80) (Table 2).

**Table 2:** Association of living alone and living alone time with hypertension.

Variables	No. of events (*n*)/incidence rate^a^	Unadjusted model	Model 1	Model 2	Model 3
		HR (95% CI)	HR (95% CI)	HR (95% CI)	HR (95% CI)
According to living arrangement					
Living with family	458 (12.4)	Reference	Reference	Reference	Reference
Living alone	115 (15.7)	1.30 (1.06–1.60)	1.37 (1.09–1.73)	1.39 (1.10–1.75)	1.42 (1.11–1.80)
According to living alone time					
Living with family	458 (12.4)	Reference	Reference	Reference	Reference
Quartile 1 (0–6.1 years)^b^	28 (16.3)	1.60 (1.09–2.34)	1.65 (1.10–2.46)	1.71 (1.14–2.55)	1.76 (1.16–2.66)
Quartile 2 (6.1–10.6 years)^b^	34 (18.9)	1.40 (0.99–1.98)	1.47 (1.01–2.12)	1.51 (1.05–2.19)	1.56 (1.07–2.29)
Quartile 3 (10.6–19.3 years) ^b^	26 (18.4)	1.55 (1.04–2.30)	1.65 (1.09–2.48)	1.59 (1.05–2.40)	1.66 (1.08–2.55)
Quartile 4 (≥19.3 years)^b^	19 (11.4)	1.00 (0.63–1.59)	1.04 (0.65–1.66)	1.03 (0.64–1.65)	1.09 (0.67–1.76)
P for trend^c^		0.173	0.201	0.230	0.163

HR, hazard ratio; CI, confidence interval. Model 1 was adjusted for age, education, residence, marital status, activities of daily living limitation, economic status, drinking status, smoking status, and regular exercise. Model 2 was further adjusted for sleep time, number of natural teeth, body mass index, diabetes, heart disease, cerebrovascular disease, and respiratory disease. Model 3 was further adjusted for the frequency of vegetable intake, frequency of fruit intake, frequency of meat intake, frequency of fish intake, and frequency of egg intake.

a Incidence rates per 100 person-years.

b Quartiles of living alone time.

c Test for trend based on the variable containing the median value for each quartile.

The crude IR of hypertension was higher among older men in the first, second, and third quartiles of time spent living alone, as compared to those living with family (Table 2). The unadjusted model revealed that, when compared to participants living with family, individuals in the first and third quartiles of time spent living alone exhibited an increased risk of hypertension. In both model 1 and model 2, those in the first, second, and third quartiles of living alone time showed an elevated risk of hypertension. In model 3, we further adjusted for the frequency of vegetable intake, fruit intake, meat intake, fish intake, and egg intake. We found that the association between living alone time and hypertension displayed a consistent trend (Table 2). To be specific, the multivariable-adjusted HRs with 95% CIs for the first, second, and third quartiles of living alone time were 1.76 (1.16, 2.66), 1.56 (1.07, 2.29), and 1.66 (1.08, 2.55), respectively (Table 2). Interestingly, there was no significant association observed between the fourth quartile of living alone time and the risk of hypertension (Table 2).

**Figure 2:**
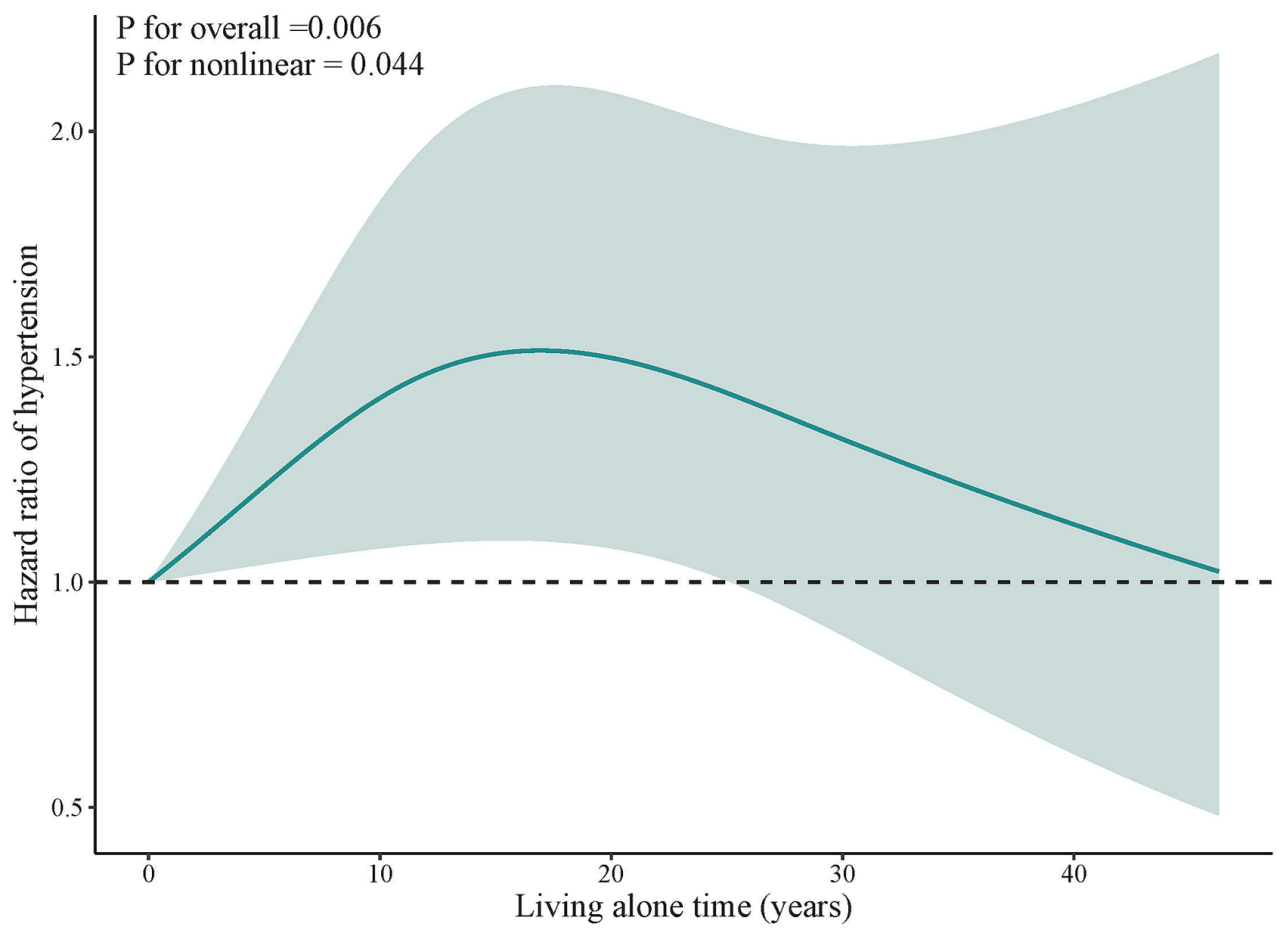
Dose–response association between living alone time and hypertension. Solid blue lines are multivariable-adjusted hazard ratios, with shaded areas showing 95% confidence intervals derived from restricted cubic spline regressions with three knots. Multivariate models were adjusted for baseline age, education, residence, marital status, activities of daily living limitation, economic status, drinking status, smoking status, regular exercise, sleep time, number of natural teeth, body mass index, diabetes, heart disease, cerebrovascular disease, respiratory disease, frequency of vegetable intake, frequency of fruit intake, frequency of meat intake, frequency of fish intake, and frequency of egg intake.

Using restricted cubic splines, we observed that the risk of hypertension increases for individuals with a duration of living alone between 0 years and 25.1 years (Figure 2). However, living alone for more than 25.1 years is not associated with hypertension.

### Subgroup analyses

**Figure 3:**
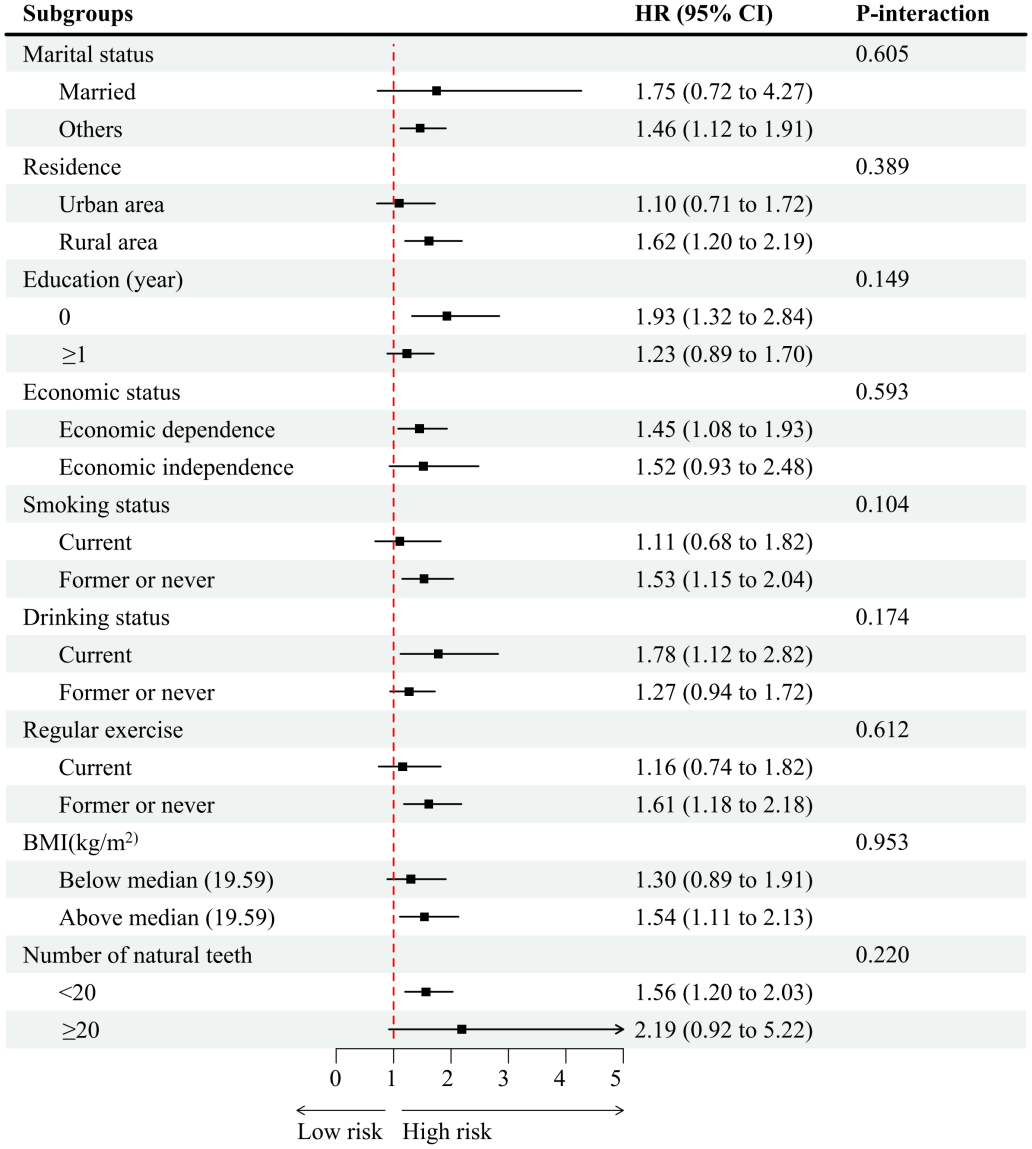
Association of living arrangements with hypertension stratified by participant characteristics. Each stratification controlled for all factors (age, education, residence, marital status, activities of daily living limitation, economic status, drinking status, smoking status, regular exercise, sleep time, number of natural teeth, body mass index, diabetes, heart disease, cerebrovascular disease, respiratory disease, frequency of vegetable intake, frequency of fruit intake, frequency of meat intake, frequency of fish intake, and frequency of egg intake) except the stratification factor itself. HR, hazard ratio; CI, confidence interval.

Stratified and interaction analyses were performed to more comprehensively assess the relationship between living alone and the risk of hypertension across different subgroups. None of the variables, such as education, residence, marital status, economic status, drinking status, smoking status, regular exercise, ADL limitation, number of natural teeth, and BMI, showed significant modification of the association between living alone and hypertension (Figure 3).

### Sensitivity analyses

The sensitivity analyses demonstrated that the associations between living alone and living alone time and hypertension remained consistent with our main analysis, even after (1) excluding 47 participants with missing data (*n* = 1962, Supplementary Tables S3, S4); (2) excluding individuals who developed hypertension within one year after the baseline survey (*n* = 1,647, Supplementary Tables S3, S4); (3) excluding 218 participants who had pre-existing diabetes, heart disease, or cerebrovascular disease at baseline (*n* = 1791, Supplementary Tables S3, S4), and (4) adjusting the weights (*n* = 2009, Supplementary Tables S3, S4).

## Discussion

The findings of this cohort study offer evidence of a significant association between living alone and the risk of hypertension in older men over 80 years of age. Our results revealed that older men living alone faced a 42% higher risk of hypertension compared to those living with family, even after adjusting for potential confounding variables. To explore the relationship further, we stratified the time spent living alone into quartiles and observed distinct patterns. The first quartile exhibited the highest risk of hypertension, followed by the third quartile, while the second quartile fell in between. Interestingly, the fourth quartile of living alone time showed no significant association with hypertension risk. This study contributes valuable insights to the existing literature, as it specifically focuses on a population often underrepresented in research studies: older men over 80 years of age. By examining this unique age group, we shed light on the distinct challenges and risk factors associated with hypertension in older men living alone.

The findings are in line with previous studies indicating that living alone was associated with a higher risk of hypertension among older adults (16, 17). For instance, our earlier study demonstrated that older adults aged 65 years or older who lived alone at baseline or continued living alone during follow-up experienced an increased risk of hypertension (16). Similarly, a cross-sectional study reported that individuals aged 65 years or older living with others had a lower risk of hypertension compared to those living alone (17). Notably, this current study’s primary focus lies on older men aged over 80 years, a unique subgroup facing distinct challenges and health needs in comparison to younger cohorts explored in previous research. While our latest study also explored the association between living alone and hypertension among older adults in China as our previous study, the key differences and contributions of our current study lie in the additional analysis focusing on the association between the duration of time spent alone and the incidence of hypertension among older Chinese men to extend and deepen this investigation. In contrast to our results, two other cross-sectional studies have shown that middle-aged and older adults (≥45 years old) living alone exhibited a reduced risk of hypertension compared to those cohabitating with others (18, 19). The inconsistencies observed in the literature may arise due to several factors. Firstly, age-related physiological changes, comorbidities, and lifestyle factors could collectively contribute to diverse associations between living arrangements and hypertension risk. Secondly, the influence of cultural norms, social support systems, and healthcare accessibility may vary among different populations, leading to discrepancies in the observed results. Finally, it is essential to recognize that cross-sectional studies inherently possess limitations, including selection bias and the inability to establish causality. Therefore, the observed inconsistencies in results may be attributed to age differences, socioeconomic status, social support, cultural variations, and study design. Unfortunately, our study was confined to male participants aged over 80 during the data analysis phase, thereby hindering a direct comparison between males and females. Nevertheless, our subgroup analysis, stemming from a prior study, revealed no discernible link between living alone and hypertension risk in women aged over 65 (16). Existing evidence also indicates that men living alone tend to experience isolation and depression earlier than women (33), potentially due to their limited social connections (34). While our research concentrated on a specific demographic subset of older men, we posit that delving into and comparing these patterns between genders could yield deeper insights into the multifaceted impacts of solitary living on the health outcomes of older adults in future studies.

Furthermore, our study suggests a potential association between living alone time and hypertension risk, although it may not exhibit a clear hierarchical or linear pattern. Our analysis indicates that the time spent alone could have an impact on hypertension development. The increased risk observed in the first, second, and third quartiles of living alone time may be attributed to various factors. Older adults who live alone may experience reduced social engagement and limited opportunities for social interaction, leading to feelings of loneliness and isolation (35, 36). This social isolation can contribute to chronic stress, which, in turn, can lead to the dysregulation of physiological systems, including the cardiovascular system (37). Moreover, individuals living alone may face challenges in maintaining a healthy lifestyle. In a systematic review conducted by Hanna and Collins (38), it was observed that individuals who lived alone often displayed an unhealthy dietary pattern in comparison to those living with family. This pattern was marked by a reduced diversity in the consumption of fruits, vegetables, meat, seafood, and eggs (38). Additional research has suggested that individuals who live alone may face limitations in accessing opportunities for engaging in physical activity, as well as a reduction in available social support for both the initiation and continuation of healthy behaviors (39–41). Our study revealed that individuals who lived alone were more likely to abstain from regular exercise and have an insufficient consumption of fruits, vegetables, meat, fish, and eggs, in contrast to participants residing with their families. Poor dietary habits and lack of physical activity are well-established risk factors for hypertension. Our findings also reveal that individuals living alone exhibited higher financial reliance compared to those living with family. Building upon previous studies that have highlighted the connection between low socioeconomic status and elevated blood pressure (42). The increased financial dependence among those living alone may underscore the significance of socioeconomic factors as contributors to hypertension in older Chinese men. The combination of these factors may increase the susceptibility to hypertension in older men living alone.

Furthermore, our investigation revealed that individuals in the first quartile of living alone time displayed the greatest susceptibility to hypertension. We speculate that recent shifts towards solitary living might signify substantial life adjustments, such as coping with loss or relocating to a new environment. Based on a prior study, the likelihood of experiencing depressive symptoms was found to substantially increase during the initial, second, and third years of living alone in comparison to residing with others (33). Notably, the greatest susceptibility to depression was observed within the initial year of living alone (33). It is conceivable that individuals who have been living alone for shorter periods encounter elevated stress levels due to the newness of their circumstances and the absence of sufficient social support networks.

Our analysis unveiled that durations of living alone exceeding 25.1 years did not demonstrate a statistically significant correlation with the risk of hypertension. Moreover, the lack of a significant relationship between hypertension risk and the fourth quartile of living alone time prompts intriguing inquiries into potential protective mechanisms. Individuals in the fourth quartile may have developed effective coping strategies, resilience, or adaptive behaviors that mitigate the adverse effects of prolonged solitary living. These individuals might maintain social connections through virtual means, participate in community events, or receive regular support from healthcare professionals or family members, counteracting the negative impact of living alone. Another plausible explanation for the lack of a significant association in the fourth quartile could be the “healthy survivor” effect. Participants who manage to maintain good health and well-being despite prolonged living alone might possess certain characteristics or lifestyle choices that protect them from hypertension development. These individuals may have a more robust support system, adhere to healthier dietary patterns, or engage in regular physical activity, which could attenuate the potential adverse effects of living alone on hypertension risk.

These findings indicated that public health initiatives should consider the potential risk posed by shorter-term social isolation and the importance of fostering social support networks to promote cardiovascular well-being. Regular blood pressure monitoring, lifestyle modifications (e.g., healthy diet, physical activity), and social engagement programs should be considered as part of comprehensive hypertension management strategies for this population. Further research is warranted to elucidate the underlying mechanisms through which living alone and the duration of living alone contribute to hypertension risk. The inclusion of objective measures such as social network size, social support scales, and biomarkers of stress and blood pressure regulation would enhance the precision of future investigations. Additionally, intervention studies evaluating the effectiveness of social support interventions in reducing hypertension risk among older adults living alone are needed.

## Strengths and limitations

The strengths of our study include its prospective design, representative sample, and focus on a specific subgroup of older men over 80 years of age. The adjustment for potential confounders further strengthens the validity of the findings. However, several limitations should be considered. First, the study focused exclusively on older men over 80 years of age, limiting generalizability to other populations. Second, living alone time was self-reported and subject to recall bias. Third, living arrangements were assessed at baseline only, and changes in living situations during the follow-up period were not accounted for. In addition, the study relied on blood pressure measurements taken at specific time points, which may not capture the variability of blood pressure over time. Finally, unmeasured confounders, such as genetic predisposition and social support networks, may have influenced the observed associations.

## Conclusion

The findings of this study indicate that among the oldest-old men, living alone is associated with an increased risk of hypertension. The duration of living alone appears to play a role in the magnitude of this risk, with the highest risk observed in the early and intermediate periods of living alone. However, the risk does not further increase in the long term. This finding suggests that the impact of living alone on hypertension risk may attenuate over time. Overall, these findings highlight the potential importance of social support and companionship in reducing hypertension risk, especially during the initial stages of living alone. Nonetheless, further research is warranted to explore the mechanisms underlying the association between living alone and hypertension risk, as well as to determine if there are specific time frames during which interventions can effectively mitigate this increased risk.

## Data availability statement

The raw data supporting the conclusions of this article will be made available by the authors, without undue reservation.

## Ethics statement

The studies involving humans were approved by the studies involving human participants were reviewed and approved by the Ethics Committee of Peking University (IRB00001052-13074). The studies were conducted in accordance with the local legislation and institutional requirements. The participants provided their written informed consent to participate in this study.

## Author contributions

XW: Conceptualization, Data curation, Formal analysis, Investigation, Methodology, Writing – original draft. MD: Conceptualization, Data curation, Funding acquisition, Investigation, Methodology, Project administration, Resources, Software, Supervision, Writing – review & editing. JX: Conceptualization, Data curation, Investigation, Methodology, Software, Supervision, Validation, Visualization, Writing – review & editing.
