# Supplementary material for: Association of living alone and living alone time with hypertension among Chinese men aged 80 years and older: a cohort study
Source: Front Public Health. 2024 Jan 5;11:1274955. doi: 10.3389/fpubh.2023.1274955 (PMC10796616; doi:10.3389/fpubh.2023.1274955)
Supplement: Supplementary file 1 [file Table_1.docx]

**Supplementary Table 1**

The numbers (percentage) of the missing variables ^a^.

| Characteristics | Number (%) with missing data |
| --- | --- |
| Sleep time | 10 (0.5) |
| Number of natural teeth | 6 (0.3) |
| Body Mass Index | 27 (1.3) |

Notes: ^a^ List only the variables with missing data.

**Supplementary Table 2**

Baseline participant characteristics stratified by the onset of hypertension during follow-up

| Characteristics | Overall  (n = 2,009) | Non-hypertension  (n = 1,436) | Hypertension  (n = 573) | P value |
| --- | --- | --- | --- | --- |
| Age (years), median (IQR) | 90.00 (85.00, 95.00) | 91.00 (86.00, 96.00) | 89.00 (84.00, 93.00) | <0.001 |
| Rural area, no. (%) | 1260 (62.7) | 911 (63.4) | 349 (60.9) | 0.313 |
| Married, no. (%) | 739 (36.8) | 494 (34.4) | 245 (42.8) | 0.001 |
| Education (year), no. (%) |  |  |  | 0.003 |
| 0 | 912 (45.4) | 684 (47.6) | 228 (39.8) |  |
| 1-6 | 851 (42.4) | 576 (40.1) | 275 (48.0) |  |
| >6 | 246 (12.2) | 176 (12.3) | 70 (12.2) |  |
| Economic independence, no. (%) | 538 (26.8) | 363 (25.3) | 175 (30.5) | 0.019 |
| Smoking status, no. (%) |  |  |  | 0.232 |
| Never | 868 (43.2) | 631 (43.9) | 237 (41.4) |  |
| Current | 585 (29.1) | 423 (29.5) | 162 (28.3) |  |
| Former | 556 (27.7) | 382 (26.6) | 174 (30.4) |  |
| Drinking status, no. (%) |  |  |  | 0.676 |
| Never | 1,015 (50.5) | 729 (50.8) | 286 (49.9) |  |
| Current | 555 (27.6) | 389 (27.1) | 166 (29.0) |  |
| Former | 439 (21.9) | 318 (22.1) | 121 (21.1) |  |
| Regular exercise, no. (%) |  |  |  | 0.003 |
| Never | 1,118 (55.6) | 817 (56.9) | 301 (52.5) |  |
| Current | 608 (30.3) | 404 (28.1) | 204 (35.6) |  |
| Former | 283 (14.1) | 215 (15.0) | 68 (11.9) |  |
| ADL limitation, no.(%) | 366 (18.2) | 308 (21.4) | 58 (10.1) | <0.001 |
| Sleep time (h), no. (%) |  |  |  | 0.149 |
| <6 | 191 (9.5) | 139 (9.7) | 52 (9.1) |  |
| 6–9 | 927 (46.1) | 643 (44.8) | 284 (49.6) |  |
| ≥9 | 891 (44.4) | 654 (45.5) | 237 (41.4) |  |
| Natural tooth number, no. (%) |  |  |  | 0.001 |
| 0-9 | 1,473 (73.3) | 1,083 (75.4) | 390 (68.1) |  |
| 10-20 | 324 (16.1) | 222 (15.5) | 102 (17.8) |  |
| ≥20 | 212 (10.6) | 131 (9.1) | 81 (14.1) |  |
| BMI (kg/m2), no. (%) |  |  |  | 0.025 |
| Underweight (<18.5) | 710 (35.3) | 535 (37.3) | 175 (30.5) |  |
| Normal (18.5–24) | 1,118 (55.6) | 778 (54.2) | 340 (59.3) |  |
| Overweight (24–28) | 152 (7.6) | 101 (7.0) | 51 (8.9) |  |
| Obese (≥28) | 29 (1.4) | 22 (1.5) | 7 (1.2) |  |
| Intake of fruit, no. (%) |  |  |  | 0.285 |
| Daily | 240 (11.9) | 175 (12.2) | 65 (11.3) |  |
| Quite often | 432 (21.5) | 304 (21.2) | 128 (22.3) |  |
| Occasionally | 807 (40.2) | 563 (39.2) | 244 (42.6) |  |
| Rarely or none | 530 (26.4) | 394 (27.4) | 136 (23.7) |  |
| Intake of vegetables, no. (%) |  |  |  | 0.181 |
| Daily | 1,136 (56.5) | 802 (55.8) | 334 (58.3) |  |
| Quite often | 585 (29.1) | 414 (28.8) | 171 (29.8) |  |
| Occasionally | 229 (11.4) | 172 (12.0) | 57 (9.9) |  |
| Rarely or none | 59 (2.9) | 48 (3.3) | 11 (1.9) |  |
| Intake of meat, no. (%) |  |  |  | 0.464 |
| Daily | 600 (29.9) | 414 (28.8) | 186 (32.5) |  |
| Weekly | 774 (38.5) | 560 (39.0) | 214 (37.3) |  |
| Monthly | 223 (11.1) | 162 (11.3) | 61 (10.6) |  |
| Occasionally | 204 (10.2) | 144 (10.0) | 60 (10.5) |  |
| Rarely or none | 208 (10.4) | 156 (10.9) | 52 (9.1) |  |
| Intake of fish, no. (%) |  |  |  | 0.126 |
| Daily | 141 (7.0) | 99 (6.9) | 42 (7.3) |  |
| Weekly | 636 (31.7) | 432 (30.1) | 204 (35.6) |  |
| Monthly | 380 (18.9) | 274 (19.1) | 106 (18.5) |  |
| Occasionally | 372 (18.5) | 272 (18.9) | 100 (17.5) |  |
| Rarely or none | 480 (23.9) | 359 (25.0) | 121 (21.1) |  |
| Intake of egg, no. (%) |  |  |  | 0.300 |
| Daily | 683 (34.0) | 508 (35.4) | 175 (30.5) |  |
| Weekly | 717 (35.7) | 508 (35.4) | 209 (36.5) |  |
| Monthly | 254 (12.6) | 175 (12.2) | 79 (13.8) |  |
| Occasionally | 193 (9.6) | 134 (9.3) | 59 (10.3) |  |
| Rarely or none | 162 (8.1) | 111 (7.7) | 51 (8.9) |  |
| Diabetes, no. (%) | 35 (1.7) | 26 (1.8) | 9 (1.6) | 0.855 |
| Heart disease, no. (%) | 102 (5.1) | 77 (5.4) | 25 (4.4) | 0.419 |
| Stroke or CVD, no. (%) | 107 (5.3) | 76 (5.3) | 31 (5.4) | 1.000 |
| Respiratory disease, no. (%) | 273 (13.6) | 202 (14.1) | 71 (12.4) | 0.359 |
| Cancer, no. (%) | 10 (0.5) | 7 (0.5) | 3 (0.5) | 1.000 |

Abbreviations: ADL, activity of daily living; BMI, Body Mass Index. Notes: Differences in characteristics were compared using the χ^2^ test for categorical variables and the Mann-Whitney *U* test for continuous variables.

**Supplementary Table 3**

Sensitive analysis of the association between living arrangements and hypertension.

| Variables | Unadjusted model | Model 1 | Model 2 | Model 3 |
| --- | --- | --- | --- | --- |
|  | HR (95% CI) | HR (95% CI) | HR (95% CI) | HR (95% CI) |
| Complete cases (n = 1962) | | | |  |
| Living with family | Reference | Reference | Reference | Reference |
| Living alone | 1.32 (1.07-1.62) | 1.37 (1.09-1.73) | 1.38 (1.10-1.75) | 1.41 (1.11-1.80) |
| Excluded participants who suffered from hypertension in the first 1 year of follow-up (n = 1647) | | | | |
| Living with family | Reference | Reference | Reference | Reference |
| Living alone | 1.30 (1.05-1.62) | 1.37 (1.08-1.75) | 1.38 (1.09-1.77) | 1.44 (1.12-1.86) |
| Excluded participants who had diabetes, heart disease, or cerebrovascular disease (n = 1791) | | | | |
| Living with family | Reference | Reference | Reference | Reference |
| Living alone | 1.29 (1.04-1.59) | 1.35 (1.06-1.71) | 1.35 (1.06-1.72) | 1.39 (1.08-1.79) |
| After adjusting the weights (n = 2009) | | | | |
| Living with family | Reference | Reference | Reference | Reference |
| Living alone | 1.30 (1.06-1.60) | 1.38 (1.09-1.73) | 1.39 (1.10-1.75) | 1.42 (1.11-1.81) |

Abbreviations: HR, hazard ratio; CI, confidence interval.

Notes: Model 1 was adjusted for age, education, residence, marital status, activities of daily living limitation, economic status, drinking status, smoking status, and regular exercise.

Model 2 was further adjusted for sleep time,[number of natural teeth, body mass index](javascript:;), diabetes, heart disease, cerebrovascular disease, and respiratory disease.

Model 3 was further adjusted for the frequency of vegetable intake, frequency of fruit intake, frequency of meat intake, frequency of fish intake, and frequency of egg intake.

**Supplementary Table 4**

Sensitive analysis of the association between living alone time and hypertension.

| Variables | Unadjusted model | Model 1 | Model 2 | Model 3 |
| --- | --- | --- | --- | --- |
|  | HR (95% CI) | HR (95% CI) | HR (95% CI) | HR (95% CI) |
| Complete cases (n = 1930) | | | |  |
| Living with family | Reference | Reference | Reference | Reference |
| Quartile 1 (0-6.1 years) ^a^ | 1.68 (1.14-2.46) | 1.71 (1.15-2.56) | 1.80 (1.2-2.69) | 1.81 (1.20-2.74) |
| Quartile 2 (6.1-10.6 years) ^a^ | 1.43 (1.00-2.03) | 1.47 (1.01-2.13) | 1.51 (1.04-2.20) | 1.57 (1.06-2.31) |
| Quartile 3 (10.6-19.3 years) ^a^ | 1.54 (1.04-2.29) | 1.61 (1.07-2.43) | 1.56 (1.03-2.36) | 1.63 (1.06-2.52) |
| Quartile 4 (≥19.3 years) ^a^ | 1.00 (0.63-1.58) | 1.02 (0.63-1.63) | 1.00 (0.62-1.61) | 1.06 (0.65-1.73) |
| Excluded participants who suffered from hypertension in the first 1 year of follow-up (n = 1621) | | | | |
| Living with family | Reference | Reference | Reference | Reference |
| Quartile 1 (0-6.1 years) ^a^ | 1.52 (1.01-2.30) | 1.55 (1.00-2.38) | 1.60 (1.03-2.47) | 1.70 (1.09-2.66) |
| Quartile 2 (6.1-10.6 years) ^a^ | 1.37 (0.95-1.98) | 1.44 (0.98-2.12) | 1.47 (1.00-2.17) | 1.53 (1.03-2.29) |
| Quartile 3 (10.6-19.3 years) ^a^ | 1.58 (1.05-2.39) | 1.69 (1.10-2.59) | 1.64 (1.06-2.52) | 1.78 (1.13-2.79) |
| Quartile 4 (≥19.3 years) ^a^ | 1.05 (0.65-1.68) | 1.09 (0.67-1.77) | 1.08 (0.66-1.76) | 1.18 (0.71-1.95) |
| Excluded participants who had diabetes, heart disease, or cerebrovascular disease (n =1759) | | | | |
| Living with family | Reference | Reference | Reference | Reference |
| Quartile 1 (0-6.1 years) ^a^ | 1.64 (1.10-2.46) | 1.67 (1.09-2.55) | 1.71 (1.12-2.61) | 1.88 (1.21-2.91) |
| Quartile 2 (6.1-10.6 years) ^a^ | 1.37 (0.95-1.96) | 1.43 (0.98-2.10) | 1.47 (1.00-2.15) | 1.51 (1.01-2.24) |
| Quartile 3 (10.6-19.3 years) ^a^ | 1.47 (0.96-2.23) | 1.54 (1.00-2.37) | 1.48 (0.95-2.29) | 1.48 (0.94-2.34) |
| Quartile 4 (≥19.3 years) ^a^ | 1.03 (0.65-1.64) | 1.07 (0.66-1.72) | 1.04 (0.65-1.68) | 1.11 (0.68-1.81) |
| After adjusting the weights (n = 1976) | | | | |
| Living with family | Reference | Reference | Reference | Reference |
| Quartile 1 (0-6.1 years) ^a^ | 1.60 (1.09-2.34) | 1.65 (1.10-2.46) | 1.71 (1.14-2.55) | 1.76 (1.16-2.66) |
| Quartile 2 (6.1-10.6 years) ^a^ | 1.40 (0.99-1.98) | 1.47 (1.01-2.12) | 1.51 (1.05-2.19) | 1.57 (1.07-2.30) |
| Quartile 3 (10.6-19.3 years) ^a^ | 1.55 (1.04-2.30) | 1.65 (1.10-2.49) | 1.59 (1.05-2.41) | 1.67 (1.08-2.57) |
| Quartile 4 (≥19.3 years) ^a^ | 1.00 (0.63-1.59) | 1.04 (0.65-1.67) | 1.03 (0.64-1.65) | 1.09 (0.67-1.77) |

Abbreviations: HR, hazard ratio; CI, confidence interval.

Notes: Model 1 was adjusted for age, education, residence, marital status, activities of daily living limitation, economic status, drinking status, smoking status, and regular exercise.

Model 2 was further adjusted for sleep time,[number of natural teeth, body mass index](javascript:;), diabetes, heart disease, cerebrovascular disease, and respiratory disease.

Model 3 was further adjusted for the frequency of vegetable intake, frequency of fruit intake, frequency of meat intake, frequency of fish intake, and frequency of egg intake.

^a^ Quartiles of living alone time.
